# Intraoperative evaluation of hepatic artery blood flow during pancreatoduodenectomy (HEPARFLOW): Protocol of an exploratory study

**DOI:** 10.1016/j.isjp.2020.03.003

**Published:** 2020-04-04

**Authors:** Mohammed Al-Saeedi, Leonie Frank-Moldzio, Miriam Klauß, Philipp Mayer, Tom Bruckner, Elias Khajeh, Mohammad Golriz, Arianeb Mehrabi, Phillip Knebel, Markus K. Diener, Markus W. Büchler, Oliver Strobel

**Affiliations:** a Department of General, Visceral and Transplant Surgery, University of Heidelberg, Germany; b Department of Diagnostic and Interventional Radiology, University Hospital Heidelberg, Germany; c Institute of Medical Biometry and Informatics, University of Heidelberg, Germany; d The Study Center of the German Surgical Society (SDGC), University of Heidelberg, Germany

**Keywords:** CT, computed tomography, ISGLS, International Study Group of Liver Surgery, CRF, case report form, Hepatic artery blood flow, Pancreatoduodenectomy, Study protocol

## Abstract

**Introduction:**

Pancreatoduodenectomy is the treatment of choice for a range of benign and malignant diseases. The pancreatic head must be separated from its supplying vessels, especially the gastroduodenal artery, during this operation. However, dissection of the gastroduodenal artery can disturb blood supply to the liver and result in liver ischemia. There is currently no well-established algorithm to evaluate and ensure sufficient blood flow in patients with altered hepatic artery blood flow. To address this important issue, this study aims to establish a basis for assessing liver blood supply during pancreatoduodenectomy. Furthermore, factors influencing arterial blood flow and related postoperative complications will be evaluated.

**Methods and analysis:**

The HEPARFLOW study is a single institutional single-arm prospective exploratory observational clinical trial. All consecutive patients undergoing elective partial or total pancreatoduodenectomy will be screened for inclusion until 100 patients are enrolled. Blood flow in the proper hepatic artery, gastroduodenal artery, portal vein, and additional vessels supplying the liver will be measured during pancreatoduodenectomy using Doppler flowmetry. All patients will be followed up for 90 days after surgery. At each visit, standard clinical data, postoperative complications and mortality will be recorded.

**Discussion:**

This will be the first study to prospectively assess intraoperative flow rates of the hepatic artery and portal vein to evaluate liver blood supply during pancreatoduodenectomy. The preoperative and intraoperative factors influencing blood flow in the hepatic arteries will be identified. This study may also reveal the hemodynamic and clinical relevance of a compression of the celiac axis during pancreatoduodenectomy.

**Ethics and dissemination:**

This study was approved by the Ethics Committee of the University of Heidelberg (S-073/2018). The results will be published in a peer-reviewed journal and will be presented at medical meetings.

## Highlights

• Assessment of flow rates of the hepatic artery during pancreatoduodenectomy.
• Identification of pre- and intraoperative factors influencing liver blood flow.
• Relevance of a celiac axis stenosis by pancreatoduodenectomy.

## 1 Background

Pancreatoduodenectomy remains the oncological treatment of choice for a range of malignant diseases, including ampullary carcinoma, distal cholangiocarcinoma, progressive intraductal papillary mucinous neoplasm, pancreatic adenocarcinoma, and benign diseases such as chronic pancreatitis and biliary duct adenoma [1], [2]. From the oncologic point of view, tumor infiltration might indicate extended arterial or venous resection [3], [4], [5], which require the pancreas to be separated from its supplying vessels, in particular the gastroduodenal artery.

The gastroduodenal artery is a branch of the common hepatic artery along with the proper hepatic artery, which provides most of the liver’s blood supply. Thus, dissection of the gastroduodenal artery disturbs blood supply to the liver during pancreatoduodenectomy. This is usually compensated without postoperative complication or liver ischemia. However, in some patients undergoing extended resections with anatomical variations or unknown coeliac axis stenosis, inadequate blood flow through the hepatic artery can prevent liver perfusion [6], [7]. This is important in pancreatoduodenectomy patients because the gastroduodenal artery is lost, which may cause ischemic complications and postoperative liver perfusion failure [8].

To avoid this, it is highly recommended to perform the clamping test before the artery is resected, to determine whether blood supply is sufficient [7]. Here, the surgeon manually tests the pulsating flow in the proper hepatic artery after clamping the gastroduodenal artery. Such a test is, of course, subjective and depends on the attending surgeon’s perspective. Alternatively, Doppler flowmetry can quantify the expected remaining perfusion from the proper hepatic artery.

There is no well-established algorithm to assess and ensure sufficient blood flow in patients with alternated hepatic artery blood flow. Consistent intraoperative measurement of arterial and portal-venous blood flow to the liver via Doppler flowmetry can assess liver perfusion during pancreatoduodenectomy. The aim of this study is to establish a basis for evaluating liver blood supply during pancreatoduodenectomy. Furthermore, factors influencing arterial blood flow and related postoperative complications will be evaluated and identified.

## 2 Methods and analysis

### 2.1 Study design

This is a single institutional one-arm prospective exploratory observational clinical trial designed in accordance with the declaration of Helsinki. This study was initiated in April 2018 and is expected to progress for 2 years. The study protocol was registered at the German Clinical Trials Register (registration number: DRKS00014620).

### 2.2 Patient eligibility

**Fig. 1:**
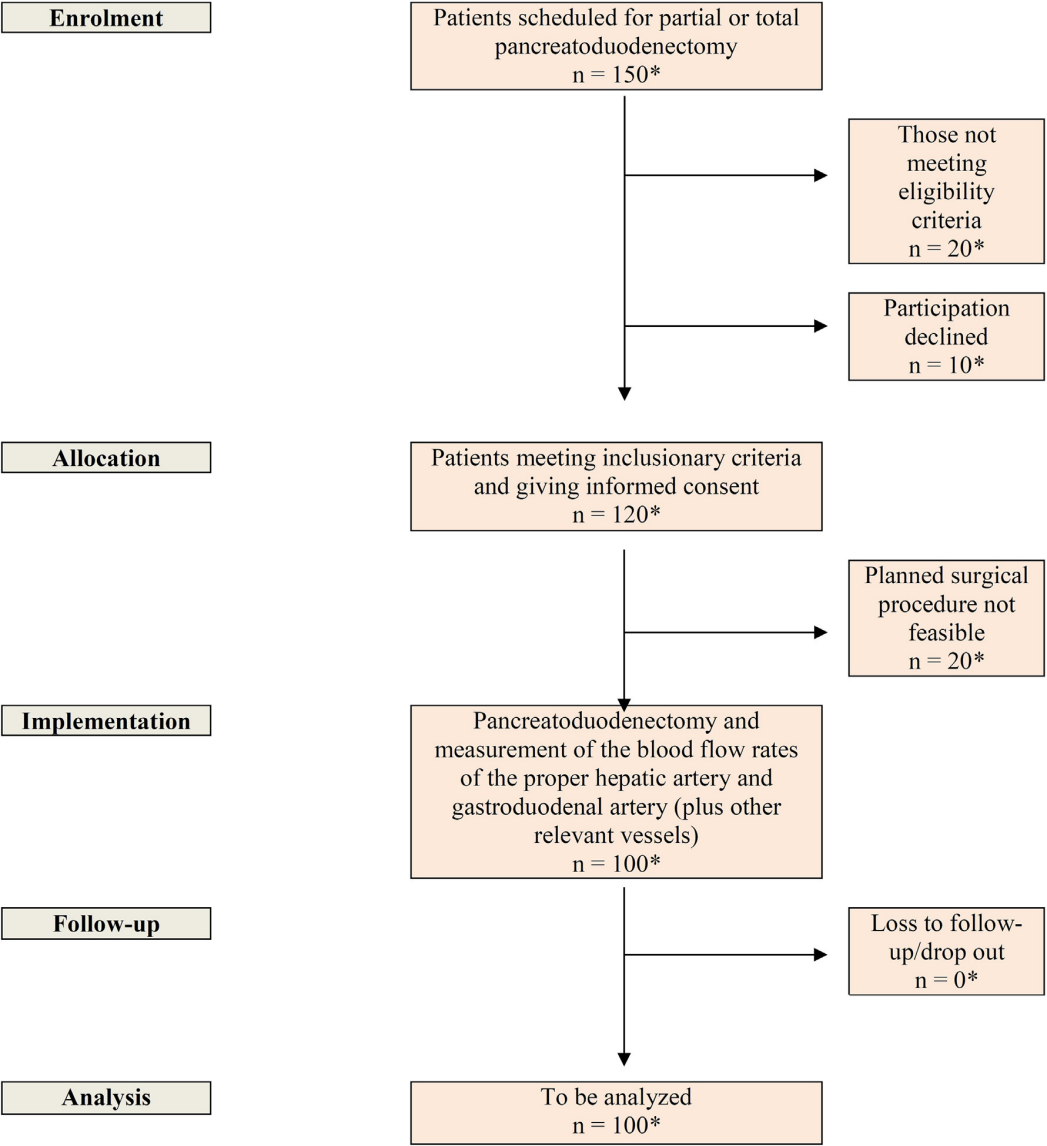
Study flow chart. *Numbers are estimated.

All consecutive patients undergoing elective pancreatoduodenectomy will be screened for inclusion until 100 patients are enrolled (Fig. 1). To account for patients who do not meet eligibility criteria, patients who refuse to participate, and intraoperative decisions against resection, it was estimated that 150 patients need to be screened.

#### 2.2.1 Inclusion and exclusion criteria

Inclusion criteria are: 1) ≥ 18 years, 2) undergoing elective open partial or total pancreatoduodenectomy, and 3) have given consent to participate in the study. Exclusion criteria are: 1) previous surgery on upper gastrointestinal organs (esophageal resection, gastric surgery, splenectomy, resection of one or more liver segments, liver transplantation, pancreas left resection, and vascular surgery of visceral vessels), 2) liver cirrhosis or other severe liver disease, and 3) bilirubin levels ≥ 15 mg/dl. In patients with pancreatic tumors, it is not always possible to predict whether resection is feasible before surgery. In these cases, an explorative laparotomy is needed to determine the appropriate surgical procedure. If extensive tumor infiltration or metastases are present, surgery cannot be performed and these patients must be excluded from the study.

### 2.3 Patient recruitment and evaluation

This study was approved by the Ethics Committee of the University of Heidelberg (S-073/2018). As presented in Fig. 1, all patients scheduled for partial or total pancreatoduodenectomy will be screened for eligibility. Preoperative assessment, including medical history, physical examination, and laboratory evaluations, will be performed according to Table 1.

**Table 1:** Preoperative parameters to be evaluated in included patients.

Demographic and baseline data	• Gender (f/m) • Age (years) • Body mass index (kg/m^2^) • ASA classification • Medication • Alcohol consumption • Nicotine intake
Comorbidity	• Cardiovascular diseases • Blood coagulation disorders • Liver diseases • Pancreas diseases • Cancer
Previous interventions	• Chemoradiotherapy • Operations
Laboratory data	• Aspartate transaminase (U/l) • Alanine aminotransferase (U/l) • Albumin (g/l) • International normalized ratio • Serum bilirubin (mg/dl) • Gamma-glutamyltransferase (U/l) • Alkaline phosphatase (U/l) • Amylase (U/l) • Leukocytes (/nl) • Creatinine (mg/dl) • Blood urea nitrogen (mg/dl) • Hemoglobin (g/dl) • Hematocrit (l/l)

#### 2.3.1 Radiological assessment

A computed tomography (CT) scan with an arterial phase will be evaluated by the attending radiologist. The focus is on anatomic variations and celiac axis stenosis, which will be classified according to the Michels classification [9]. Examples of important anatomic vessel variations are:

• Aberrant right hepatic artery originating from the superior mesenteric artery (Michels type III)
• Accessory right hepatic artery originating from the superior mesenteric artery (Michels type VI)
• Aberrant common hepatic artery originating from the superior mesenteric artery (Michels type IX)
• Aberrant left hepatic artery originating from the left gastric artery (Michels type II)
• Right and left hepatic artery originating separately from the celiac trunk

Vessel diameter will also be monitored as it may reveal dilated collaterals due to celiac axis stenosis. To calculate total liver volumes, DICOM files of preoperative CT images (portal phases) were uploaded to the local server and then evaluated using 3D volumetric software AquariusNET (TeraRecon, Inc., Foster City, CA, USA). All eligible participants who give informed consent will be operated and followed up in accordance with the routine procedures of the department of surgery at Heidelberg University Hospital.

#### 2.3.2 Intraoperative assessment

During pancreatoduodenectomy, the common/proper hepatic artery and the gastroduodenal artery (and other pancreatoduodenectomy-relevant vessels) will be routinely exposed. A DUPLEX ultrasonography probe will be placed on the artery or vein and peak systolic and diastolic velocities will be measured (these measurements require several cardiac cycles). Blood flow in the relevant vessels supplying the liver will be measured: 1) before the gastroduodenal artery is clamped (after the vessels are prepared), 2) during clamping or after division of the gastroduodenal artery, and 3) before biliodigestive anastomosis (Table 2). To assess the presence of celiac axis stenosis, the direction of the gastroduodenal artery flow will additionally be documented during the first measurement session. The negative flow shows that whether the blood flow is anterograde or retrograde. In the case of negative (retrograde) flow, the percentage of the flow will be also documented. Vital parameters including systolic and diastolic blood pressure, mean arterial pressure, pulse rate, and central venous pressure will be also documented simultaneously to blood flow rate measurements. The dosage of catecholamine administered during blood flow measurements will also be recorded. Arterial blood gas, amount of infusions, operation duration, blood loss, and intraoperative complications will also be monitored.

**Table 2:** Times of blood flow measurement during the operation.

	Time of measurement	Localization of the measuring sensor	Measuring period
I.	Before clamping the gastroduodenal artery (after preparing the vessels)	Gastroduodenal artery, proper hepatic artery, and portal vein	I (~2 min)
II.	During clamping or after division of the gastroduodenal artery	Proper hepatic artery and portal vein	
III.	Before biliodigestive anastomosis	Proper hepatic artery and portal vein	II (~1 min)

### 2.4 Outcomes

#### 2.4.1 Primary endpoint

The primary endpoint is arterial flow in the proper hepatic artery, gastroduodenal artery, and in additional liver arteries (in anatomical variations) during pancreatoduodenectomy. In order to obtain valid data that reflects blood flow in the hepatic artery during pancreatoduodenectomy, it will be important to execute the planned measurements before and during clamping or after division of the gastroduodenal artery (mL/min) for 2 minutes during each measurement session.

#### 2.4.2 Secondary endpoints

Secondary endpoints aim at the clinical relevance of blood flow rates for liver perfusion, liver-related complications and other complications (Table 3). As liver perfusion failure cannot be directly measured and quantified, it will be assessed using laboratory surrogate parameters for hepatocellular injury and liver function (Table 1, Table 3). Other post-pancreatoduodenectomy complications will be assessed according to the international accepted definitions [6], [10], [11], [12] (Table 3).

**Table 3:** Secondary endpoints of the HEPARFLOW study.

Endpoints	Definitions
Laboratory findings	Presented in Table 1
Liver volumetric assessment	Total liver volumes (cm^3^) will be evaluated using preoperative computed tomography scans
Length of intensive care unit stay	Time (days) from the day of the operation until the day of discharge from the intensive care unit
Length of hospital stay	Time (days) from the day of the operation until the day of discharge
Liver ischemia following pancreatic resection	According to Hackert et al. [6]
Posthepatectomy liver failure	Based on the ISGLS criteria [12]
Postoperative pancreatic fistula	Based on the ISGPS criteria [10]
Postoperative complications	Graded according to the Clavien–Dindo classification[11]
Mortality	Death due to any cause at any time during the follow-up period

**Fig. 2:**
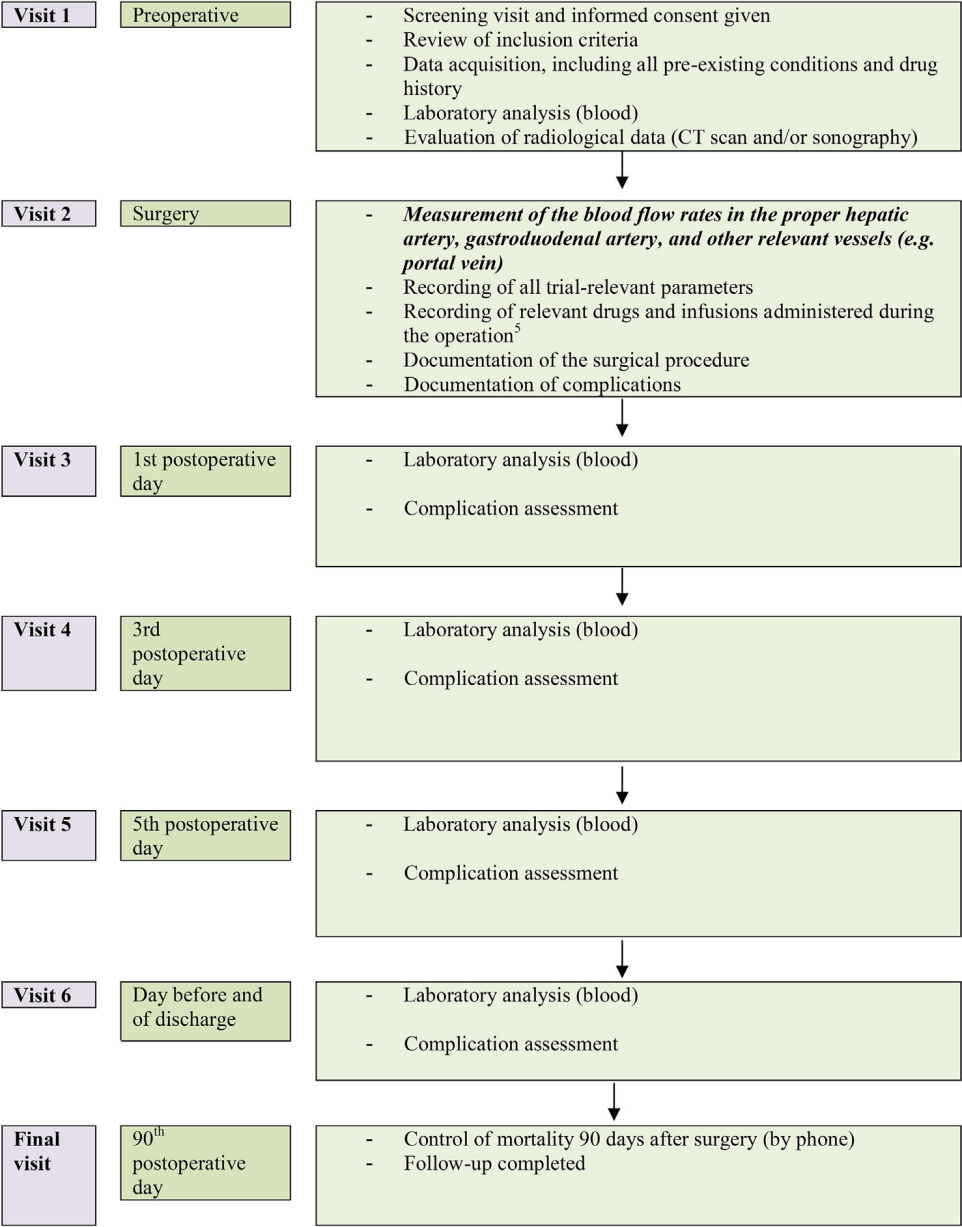
Diagram showing the perioperative visit plan of patients in the HEPARFLOW study.

The study may reveal the hemodynamic and clinical relevance of CT morphological compression of the celiac axis during pancreatoduodenectomy. To evaluate the relationship between intraoperative blood flow and liver volume, total liver volume will be calculated based on preoperative CT imaging. The postoperative course, including laboratory analysis and recorded complications on the first day, third day, fifth day, and the day before or on the day of discharge will be documented. The duration of intensive care unit and overall hospital stay will also be recorded (Fig. 2). The classification of liver ischemia following pancreatic resection will be used to demonstrate liver perfusion failure [6]. Posthepatectomy liver failure and postoperative pancreatic fistula will be diagnosed and categorized based on the International Study Group of Liver Surgery (ISGLS) [12] and the International Study Group of Pancreatic Surgery (ISGPS) [10] criteria, respectively. All postoperative complications will be documented. Complications will be graded based on the Clavien–Dindo classification [11]. Perioperative mortality will be registered and mortality will be ascertained after 90 days by phone (Fig. 2).

### 2.5 Patient and public involvement

The patients and public were not involved in the planning of this study.

### 2.6 Data collection and management

Data will be collected until 100 patients are included in the study and all HEPARFLOW measurements are completed. For each subject, a paper-based case report form (CRF) will be filled out. CRFs will be signed by the entering physician. Signed forms and data files will be sent to the trial coordinator. Data completeness will be controlled after each measurement by the investigators and collaborating team. All data will be screened and missing data will be obtained from the study database or from the participant. Each CRF will be assigned an anonymous allocation number to ensure patient confidentiality. Protocol amendments will be considered by the principal investigator.

### 2.7 Statistical design and analysis

#### 2.7.1 Sample size calculation

For this exploratory study, the sample size cannot be determined as no previous data is available to assume a difference. The aim of this exploratory pilot study is to generate preliminary data to allow sample size calculation in future studies on liver perfusion alterations due to surgery on the hepatic artery or celiac trunk. Based on the previous experiences, blood flow rate measurements from 100 patients will provide enough information about blood flow, also in patients with celiac axis stenosis caused by extrinsic compression from the median arcuate ligament. This condition affects around 10% of patients undergoing pancreatoduodenectomy according to the literature [13], [14], [15], [16].

#### 2.7.2 Statistical analysis plan

Descriptive statistics will include mean, standard deviation, minimum, median, maximum, and quartiles for continuous data, and absolute and relative frequencies for categorical data. The empirical distribution of blood flow will be visualized by histograms. Correlation coefficients will be calculated to show possible associations between blood flow and continuous baseline data and postoperative laboratory values. Possible differences between patients with and without postoperative complications will be calculated using the Wilcoxon rank-sum test. Multivariable binary logistic regression will be used to identify possible risk-factors of complications. Because the trial is explorative, all generated p-values must be treated as descriptive. Wherever appropriate, statistical graphics including histograms, scatterplots, and boxplots will be provided to visualize the findings. The significance level will be set at α < 0.05, representing 95% confidence interval.

### 2.8 Ethical and legal aspects and termination criteria

Participation in the present study is voluntary. The study protocol was approved by the independent Ethics Committee of the University of Heidelberg (S-073/2018). All patients will be informed in detail about the aim and procedures of the study. Written informed consent will be obtained from patients who agree to participate in the study. Patient data will be protected by medical confidentiality and the provisions of the Federal Data Protection Act (BDSG). Based on the European General Data Protection Regulations (EU-DSGVO), all patient data will be collected, pseudonymized and statistically analyzed. Third parties will have no access to original patient records.

Patients can withdraw from the HEPARFLOW trial at any time without explanation. The decision to withdraw a patient from the study based on the aforementioned exclusion criteria will be made by the attending physician. All data will be checked, and any missing data will be obtained from the trial database or from participants. The permission to continue follow-up and data collection will be obtained in the event of withdrawal from the study.

## 3 Discussion

HEPARFLOW is the first clinical trial designed to prospectively and objectively assess blood supply to the liver during pancreatoduodenectomy using Doppler flowmetry. All consecutive elective pancreatoduodenectomy patients will be screened for inclusion until 100 patients are enrolled. The primary aim of the current study is to assess blood flow rates in the proper hepatic artery and the gastroduodenal artery during pancreatoduodenectomy. Blood flow will be measured before and during clamping and after division of the gastroduodenal artery. After surgery, intensive care unit stay, overall hospital stay, liver ischemia, posthepatectomy liver failure, postoperative pancreatic fistula, and mortality will be recorded. The results of this trial may provide a standard range of blood flow values to the liver and to allow for a better way to assess and manage liver perfusion during pancreatoduodenectomy. This study may also identify factors influencing arterial blood flow and related complications.

## 4 Ethics approval

This protocol study was reviewed and approved by the Ethics Committee of the University of Heidelberg (S-073/2018).

## 5 Consent to participate

Not applicable

## 6 Consent for publication

Not applicable

## 7 Availability of data and materials

Not applicable

## Declaration of Competing Interest

None declared.

## Funding

This study was funded by a grant from the Heidelberg Stiftung Chirurgie (Heidelberg Foundation for Surgery).
